# Combined heterozygosity for the highly unstable variant hemoglobin Taybe, and α-thalassemia as a rare cause of hemolytic anemia

**DOI:** 10.1007/s00277-025-06578-6

**Published:** 2025-09-09

**Authors:** Saskia N. Nagel, Joaquin Brintrup, Yazan Ghannam, Andreas Stallmach, Andreas Hochhaus, Cristina Ripoll, Holger Cario, Karin G. Schrenk

**Affiliations:** 1 Abteilung für Hämatologie und Internistische Onkologie Klinik für Innere Medizin II, Universitätsklinikum Jena, Am Klinikum 1, 07747 Jena, Germany; 2 Mitteldeutsches Krebszentrum, Standort Jena, Jena, Germany; 3 Gastroenterologie, Hepatologie, Infektiologie, Interdisziplinäre Endoskopie, Klinik für Innere Medizin IV, Universitätsklinikum Jena, Jena, Germany; 4 Klinik für Kinder- und Jugendmedizin, Universitätsklinikum Ulm, Ulm, Germany

**Keywords:** Hemolysis, Hemolytic anemia, Thalassemia, Hemoglobin Taybe

## Dear editor

In addition to the well-known hemoglobin variants of thalassemia and sickle cell disease, other rare hemoglobinopathies must be considered in the differential diagnosis in patients with hemolysis [1]. Hemoglobin Taybe is an unstable α-chain hemoglobin variant, caused by in-frame deletion of three nucleotides in the α1-globin gene, with a subsequent loss of one of the two threonine residues at codons 39/40 of the α1-globin gene (HVGS nomenclature), corresponding to one of the two threonine residues at position 38/39 of the mature α1-globin protein. This deletion results in a structural abnormality that affects the α/β-globin chain contact producing a highly unstable hemoglobin. While heterozygous carriers are usually asymptomatic, homozygous patients and patients with heterozygous Hb Taybe in combination with a second α-globin mutation or deletion may be severely affected [2–4]. We report a patient, presenting heterozygosity for hemoglobin Taybe in combination with a heterozygous non-deletional α-thalassemia leading to functional loss of one α-globin gene copy.

**Fig. 1:**
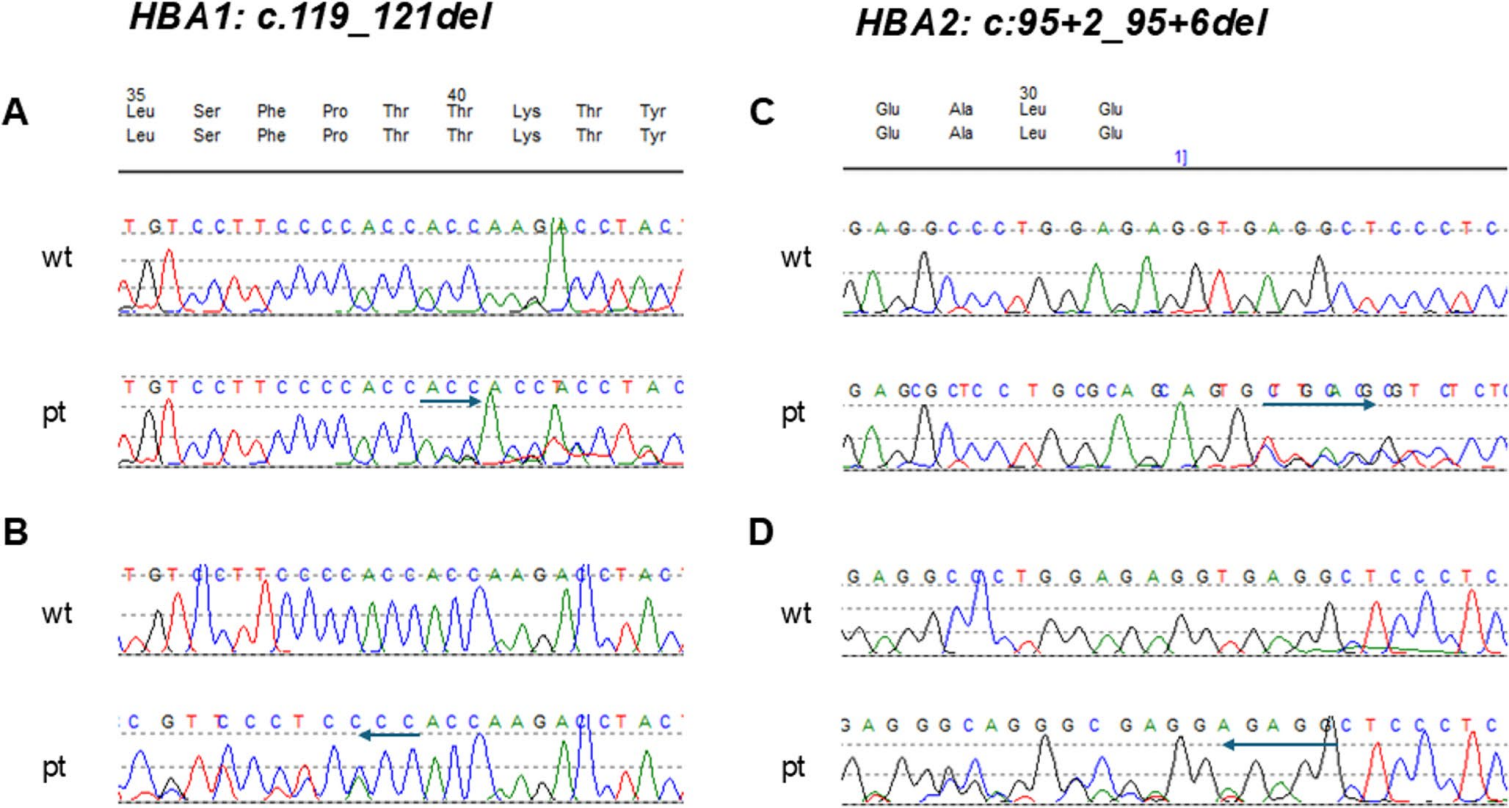
Molecular globin gene analysis

A 22-year-old Syrian male patient presented to the emergency department with painless jaundice, progressive for the last 6 months and fatigue. The patient had a history of variable jaundice since childhood. Dependent on the consumption of fruits or fatty meals, abdominal pain had occurred in the past. No medical evaluation had been performed. He had no other diseases, medication or allergies. The family history with 8 siblings was unremarkable. On clinical examination distinct splenomegaly was detected. Laboratory workup revealed Coombs negative hemolysis with hemoglobin of 6,8 mmol/l (normal range (nr) 8,7–10,9 mmol/l), lactate dehydrogenase of 15 µmol/l*s (nr < 4,2 µmol/l*s), haptoglobin of < 0,1 g/l (nr 0,3–2,0 g/l), bilirubin of 181 µmol/l (nr < 21 µmol/l) and reticulocytes of 94‰ (nr 10–20‰). The patient had an iron and vitamin B12 deficiency. Fragmentocytes were not present, hemoglobin electrophoresis revealed no abnormalities. Abdominal ultrasound demonstrated sludge of the gall bladder with no signs of intra- or extra hepatic cholestasis and splenomegaly of 21 × 8.5 cm. Because of iron deficiency, oral iron supplementation was initiated, and the patient was discharged with the recommendation of retesting. After 2 weeks, the patient was readmitted because of new abdominal pain. In addition to prior findings, elevated liver transaminases and gamma-glutamyltransferase **(**g-GT) were detected. CT-scan revealed splenomegaly with no additional pathological findings. Due to hemolysis in combination with elevated liver enzymes Wilson`s disease was considered, however, serum coeruloplasmin and copper in the 24 h urine were within normal ranges. Liver biopsy demonstrated mild cholangitis caused by repetitive obstruction, IgG4 disease or hepatic pathology was excluded. After several days the upper abdominal pain resolved. For further evaluation of hemolysis, the patient was referred to the hematology department. Further workup, including blood smears, fluorescence activated cell sorting (FACS)-analysis for spherocytosis (eosin-5-maleimide binding-test, EMA-test), and nocturnal paroxysmal hemoglobinuria (PNH) demonstrated no pathological findings. Glucose-6-phosphat dehydrogenase-, and pyruvat kinase activities were within normal ranges. However, subsequent sequencing analysis of the α1- and α2-gene (*HBA1* and *HBA2*) revealed the two heterozygous mutations *HBA1:c.119_121del* (p.Thr40del) leading to Hb Taybe and *HBA2:c95 + 2_95 + 6del*), a pentanucleotide deletion of the 5`donor site of intervening sequence (IVS) of the α2-globin gene, leading to defective splicing, responsible for non-deletional α-thalassemia (Fig. 1) [5].

Hemoglobin Taybe is a rare hemoglobin variant, which has been described mainly in Israeli-Arab individuals [2–4, 6–8]. The name Hb Taybe is derived from the town of Taybe in northern Israel, where the mutation was characterized for the first time [2, 4]. Koren et al., 2016 described 43 patients with Hb Taybe or Hb Taybe in combination with other α-gene alterations. The patients presented with variable clinical symptoms dependent on the genetic background reaching from mild to transfusion dependent hemolytic anemia [4]. This variability was revealed in the homzygous status of Hb Taybe respectively, with some siblings being more affected despite identical mutation [6–8]. Homozygous Hb Taybe may cause intrauterine hemolytic anemia and hydrops fetalis as described in triplets. However, the sister of the newborns with the same genotype only presented with mild anemia [8]. In addition, Hb Taybe in combination with α-thalassemia was observed in 4 Greek patients presenting with moderate chronic anemia [9].

Because of the presentation of upper abdominal pain our patient was initially referred to the gastroenterology department. The workup revealed elevated liver enzymes, Coombs negative hemolysis, sludge of the gall bladder, and splenomegaly. Further evaluation, including liver biopsy provided no further information. Since the abdominal pain resolved, passage of a gall stone is the most likely cause for the pain. Only the final molecular globin gene analysis unraveled the diagnosis. Although hemoglobinopathies due to unstable hemoglobin variants are rare, these mutations have to be considered in the differential diagnosis in patients with hemolytic anemia and no other apparent underlying cause. As such variants often elude detection by classic hemoglobin analysis methods, genetic analysis should be included.

Sequencing analysis reveals combined heterozygosity for two alpha-globin gene mutations, one in each *HBA1* and *HBA2*. Sequences obtained with forward primers are shown in A and C. B and D demonstrate sequences with reverse primers. Arrows indicate deleted nucleotides and sequence shift (wt, wild type; pt, patient).

## Data Availability

No datasets were generated or analysed during the current study.
